# Transcriptomic and cell wall proteomic datasets of rosettes and floral stems from five *Arabidopsis thaliana* ecotypes grown at optimal or sub-optimal temperature

**DOI:** 10.1016/j.dib.2019.104581

**Published:** 2019-09-28

**Authors:** Harold Duruflé, Philippe Ranocha, Thierry Balliau, Christophe Dunand, Elisabeth Jamet

**Affiliations:** a Laboratoire de Recherche en Sciences Végétales, Université de Toulouse, CNRS, UPS, 24 chemin de Borde Rouge, Auzeville, BP42617, 31326 Castanet Tolosan, France; b PAPPSO, GQE - Le Moulon, INRA, Univ. Paris-Sud, CNRS, AgroParisTech, Université Paris-Saclay, 91190 Gif-sur-Yvette, France

**Keywords:** *Arabidopsis thaliana*, Cell wall, Proteomics, Pyrenees ecotype, Transcriptomics

## Abstract

This article provides experimental data describing the RNA and the cell wall protein profiles of rosettes and flower stems of five *Arabidopsis thaliana* ecotypes. Four newly-described Pyrenees ecotypes [1] are analyzed in addition to the well-described and sequenced Columbia (Col) ecotype of *A. thaliana*. All five ecotypes have been grown at two different temperatures, 22 °C and 15 °C. We provide transcriptomics and cell wall proteomics data regarding (i) rosettes at the bolting stage, and (ii) floral stems at the first flower stage. These data are a valuable resource to study the adaptation of *A. thaliana* ecotypes to sub-optimal temperature growth conditions.

**Table:** Specifications Table

Subject area	Biology
More specific subject area	Transcriptomics, Cell wall proteomics
Type of data	Raw and processed data
How data was acquired	Illumina HiSeq 3000, LC-MS/MS
Data format	Tables
Experimental factors	5 ecotypes of *Arabidopsis thaliana* at 2 growth temperatures (15 and 22 °C) 2 organs (rosettes and floral stems) 3 biological replicates
Experimental features	Transcriptomics and cell wall proteomics of rosettes and floral stems
Data source location	Laboratoire de Recherche en Sciences Végétales, Université de Toulouse
Data accessibility	Resulting sequences are available at NCBI short read archive (SRA, BioProject PRJNA344545). LC-MS/MS data are deposited at PROTICdb (http://moulon.inra.fr/protic/wallomics) Cell wall proteomics data are available in this article as 2 Supplementary files.

**Table:** 

**Value of the Data** • The data provide a first level of molecular description at the transcriptomics and the cell wall proteomics levels of four newly-identified *A. thaliana* altitudinal ecotypes from the Pyrenees mountains. • The datasets include RNAseq and cell wall proteomics quantitative analyses of two organs (rosettes and stems) of five *A. thaliana* ecotypes at two growth temperatures. • The data can be used to study the impact of sub-optimal temperature growth conditions on *A. thaliana* ecotypes responses.

## 1 Data

The data provided here two different omics approaches to contribute to the understanding of the impact of sub-optimal temperature growth conditions on *A. thaliana* responses. The raw data include information about differential gene expression and cell wall protein abundance in five wild *A. thaliana* ecotypes. All the LC-MS/MS proteomics data have been deposited at PROTICdb (http://moulon.inra.fr/protic/wallomics). The RNAseq data are available at NCBI short read archive (https://www.ncbi.nlm.nih.gov/sra, BioProject PRJNA344545).

The transcriptomic and proteomic quantification datasets in rosettes and floral stems are provided in two Supplementary files including:

• Supplementary file 1: Quantitative cell wall proteomics data: proteins were extracted from purified cell walls of *A. thaliana* rosettes or floral stems after growth at 22 °C or 15 °C by saline solutions (CaCl_2_ 0.2 M and LiCl 2 M) and identified by LC-MS/MS and bioinformatics
• Supplementary file 2: Transcript levels (RNAseq data): RNA were extracted from *A. thaliana* rosettes or floral stems after growth at 22 °C or 15 °C

## 2 Experimental design, materials, and methods

### 2.1 Plant material

The experimental design and plant materials have been previously detailed [1]. In summary, five altitudinal ecotypes of *A. thaliana* were used: Grip, Hosp, Hern, Roch collected at different altitudes in the Pyrenees mountains [2], and the well-described Columbia ecotype (Col) (https://www.ebi.ac.uk/ols/ontologies/efo/terms?short_form=EFO_0005147). After being sowed in Jiffy-7® peat pellets (Jiffy International, Kristiansand, Norway), seeds were stratified for 48 h at 4 °C in darkness. Plants were grown at two different temperatures (22 °C or 15 °C), under 90 μmol photons.m^−2^.s^−1^ of light intensity under a long-day condition (16 h light/8 h dark) with 70% humidity. In total, about 20 plants from three independent batches have been used and pooled for the triplicate molecular analyses. The rosettes and floral stems were collected just before bolting (stage 5.10 [3]) and at the first flower stage of development (stage 6 [3]), respectively. The same biological triplicates were used to prepare samples for both transcriptomics and cell wall proteomics analyses.

### 2.2 Sequential extraction of proteins from purified cell wall

Cell wall purification was performed as described [4]. The sequential extraction of proteins from purified cell walls was done as described [5]. The final protein extract was lyophilized. Proteins were quantified with the CooAssay Protein Assay kit (Interchim, Montluçon, France). Typically, 0.2 g of lyophilized cell walls was used for one extraction and about 500 μg proteins were obtained.

### 2.3 Identification of proteins by LC-MS/MS

The identification of proteins extracted from cell walls was performed by LC-MS/MS at the PAPPSO proteomics platform (pappso.inra.fr/) after tryptic digestion in solution as described [6]. Parameters for MS data processing in the X!Tandem software (JACKHAMMER, 2013.6.15, www.thegpm.org/tandem/) and the X!Tandem Pipeline 3.3.4 [7] are detailed in Ref. [8]. Trypsin digestion was declared with no possible miscleavage. Only proteins identified with at least two different specific peptides in the same sample and found in at least two biological replicates were validated. Furthermore, quantification was performed on peptides with standard deviation retention times lower than 20 s.

### 2.4 Bioinformatics annotation of proteins and quantification

The prediction of sub-cellular localization of proteins was performed with the *ProtAnnDB* tool (http://www.polebio.lrsv.ups-tlse.fr/ProtAnnDB/, [9]. A protein was considered as a cell wall protein (CWP) if two bioinformatics programs predicted it as secreted, no intracellular retention signal was found and no more than one trans-membrane domain was predicted as described in Ref. [10]. Quantification was only operated for CWPs using the MassChroQ 2.2.12 software (http://pappso.inra.fr/bioinfo/masschroq/, [11]) and it was done as in Ref. [12]. Briefly, a background noise was used to replace missing data in order to facilitate the statistical analysis. This step was done differently to distinguish between validated (identification with at least two specific peptides in at least two of the three biological replicates of the ecotype/temperature combination), non-validated proteins (identification of only one specific peptide and/or in only one biological replicate) and undetectable proteins (no peptide identified in this combination). If the protein was validated, a background noise corresponding to the mean of the minimum and the first statistical quartile of the biological replicate was applied. If the protein was undetectable, a background noise of 6 (value lower than the minimum value found in the whole experiment) was applied) [12].

### 2.5 RNA sequencing (RNAseq)

Protocols used for the transcriptomic analysis have been detailed in Ref. [12]. The RNAseq data have been obtained at the Get-PlaGe platform (https://get.genotoul.fr/). Short pair-end sequencing reads generated from the Illumina platform (https://ng6.toulouse.inra.fr/) were trimmed based on the quality scores (limit: 0.05), end ambiguity (maximum allowed number of ambiguities: 2) and adaptor sequences, using the commercial CLC Genomic Workbench 8.0 software (CLC bio, Aarhus, Denmark). The online CLC protocol for trimming and assembly has been followed (https://www.qiagenbioinformatics.com/). The reads shorter than 50 bp after trimming were discarded. The TAIR 10 database has been used for the assembly process (https://www.arabidopsis.org/). The calculation of gene expression level (RPKM) has been obtained with the CLC software (settings: minimum mapped read length fraction=0.95; minimum similarity=0.98). Finally, the expression levels lower than one RPKM per gene for the total of the conditions were considered as “not expressed”.

Note that only two biological replicates could be considered for the Grip and Hosp ecotypes grown at 15 °C due to insufficient quality of the reads of the third replicates.

## Appendix A Supplementary data

Durufle et al._Supplementary File 1 Durufle et al._Supplementary File 1

Durufle et al._Supplementary File 2 Durufle et al._Supplementary File 2
